# A preliminary study on the characterization of taste and odor attributes in commercial lager and ale beers using E-sensing techniques

**DOI:** 10.3389/fnut.2026.1889963

**Published:** 2026-08-03

**Authors:** Seong Jun Hong, Mi Jeong Sung, Gwang-Ju Jang, Sang-Hee Lee

**Affiliations:** 1 Korea Food Research Institute, Wanju-Gun, Jeollabuk-do, Republic of Korea; 2 Department of Food Science and Technology, Keimyung University, Daegu, Republic of Korea

**Keywords:** ale beers, commercial canned beer, electronic nose, electronic tongue, lager beers, multivariate analysis

## Abstract

**Introduction:**

This study aimed to investigate the discrimination of taste attributes and volatile odor patterns among eight commercial canned beers representing lager and ale styles using electronic tongue (E-tongue) and electronic nose (E-nose) systems.

**Methods:**

To achieve this, five taste-related E-sensors were evaluated across the eight beer samples, and a total of 34 volatile compounds, comprising 3 acids, 4 aldehydes, 7 alcohols, 9 esters, 4 hydrocarbons, 5 heterocyclics, and 2 ketones, were tentatively identified using an E-nose internal library. Principal component analysis (PCA) and hierarchical cluster analysis (HCA) were applied to map the flavor profiles. Furthermore, partial least squares discriminant analysis (PLS-DA) was conducted to identify key flavor drivers, and a debiased sparse partial correlation (DPSC) network analysis was utilized to model the interactions between taste attributes and volatile compounds.

**Results:**

The PCA and HCA of the E-sensing datasets successfully differentiated the beer samples into distinct groups according to their taste attributes and volatile compound profiles. The PLS-DA identified six critical flavor contributors based on a variable importance in projection threshold (VIP-score) of ≥1.0, which included sweetness from the E-tongue alongside five volatile groups—acids, hydrocarbons, alcohols, heterocyclics, and aldehydes. The DPSC network analysis further elucidated these intricate interactions, establishing a stable co-occurrence framework dominated by seven key flavor clusters (ketones, sweetness, alcohols, acids, esters, heterocyclics, and hydrocarbons).

**Conclusion:**

These findings demonstrate that integrated electronic sensing platforms, combined with advanced chemometric network models, provide an effective, objective, and rapid instrumental screening approach for fingerprinting and characterizing flavor patterns in commercial lager and ale beers.

## 1 Introduction

Beer is one of the most widely consumed alcoholic beverages worldwide, accounting for approximately 80% of all types of liquor. The beer industry has remained resilient, with global production and consumption reaching approximately 1.93 billion hectoliters in 2023. In parallel, the expansion of the craft beer sector has intensified the need for rapid, objective, and low-cost quality-control methods, including E-sensing technologies, as microbreweries increasingly require real-time monitoring tools to maintain product consistency and sensory quality (1, 2). Beer is a popular alcoholic fermented beverage obtained from malt-derived worts, and it is the product of yeast-driven fermentative metabolism, which primarily converts the wort sugars into CO_2_, ethanol, and other minor compounds. These generated compounds contribute substantially to the flavor characteristics of beer found across different styles and/or categories. In fact, yeast fermentation can play a crucial role in generating various compounds, such as esters, thiols, lactones, phenolic compounds, alcohols, which contribute to the development of beer flavor attributes (3). In addition, the brewing process and conditions also contribute to its flavor attributes. Indeed, the unique flavor attributes of beer are highly influenced by different types of raw ingredients, such as variations in hop cultivar and yeast strain type (4, 5).

Taste and odor attributes play a key role in evaluating food quality and acceptability, and these characteristics are the main recognition factors of chemosensory perception in beer (6, 7). In fact, taste and odor attributes are primarily associated with taste attributes and volatile compound profiles. To evaluate the flavor characteristics and compounds in food matrices, a majority of the studies used to measure these factors through human sensory evaluation, and it can reveal overall sensory acceptability and provide information about complex sensory attributes of foods (8, 9). However, human sensory evaluation has some disadvantages, including high cost (time-consuming), a well-trained panel, and subjectivity (7).

Recently, electronic sensing (E-sensing) techniques have become highly necessary and are applied in measuring food samples. Unlike human sensory evaluation, E-sensing techniques, which include the electronic tongue (E-tongue) and electronic nose (E-nose), have several advantages, including non-destructive, low-cost, fast detection, high reproducibility, and high objectivity (10). In addition, E-sensing techniques are developed based on mimicking the sensory perception of humans to taste attributes and volatile compounds through E-tongue and E-nose, respectively (11, 12). Previously, several studies reported that the discrimination of whiskies and wines (liquors) (13) and taste attributes of low-alcohol and international pale lager were evaluated using E-tongue (14), and the classification of hops (15) and aroma profiles and quality of beer were measured using E-nose (16). As a result, E-sensing techniques can be used to distinguish and apply to the food matrix.

Accordingly, this study aimed to explore the taste and odor attributes in lager and ale beers using integrated E-tongue and E-nose platforms. While previous studies have individually evaluated beer flavors, the innovative aspect of this study lies in evaluating the synergistic combination of E-nose, E-tongue, and advanced chemometric network models as a rapid, pattern-based screening framework for commercial beers. This integrated approach is motivated by the need for a time- and cost-efficient alternative to traditional sensory panels and complex chromatographic methods for quality control in the brewing industry. In addition, the relationships between taste and volatile compounds and key compounds were identified in commercial lager and ale beers through a multivariate analysis.

## 2 Materials and methods

### 2.1 Samples

Eight beer samples (commercial canned beers) were selected according to fermentation types of beer, including four lager (A–D) and ale (E–H) beers, respectively. The samples were stored at 4 °C before the experiments. The main ingredients of commercial lager and ale beers are detailed in Table 1. For each commercial brand, biological replicates were obtained from three independent cans (*n* = 3 per brand) of the same batch, and all analytical measurements were performed in triplicate to ensure reproducibility. To provide a sensory baseline for the E-sensing measurements, the expected flavor profiles of the selected samples can be broadly categorized by their styles and specific ingredient formulations. The lager samples (A–D) represent classic bottom-fermented styles, typically characterized by a clean, crisp., and refreshing profile with subtle malt notes and mild hop bitterness. In contrast, the ale samples exhibit more diverse sensory attributes driven by top-fermentation and specific adjuncts. Samples E–G correspond to specialty or flavored ales (Belgian-style witbiers or fruit ales), which are characterized by prominent sweetness, lower bitterness, and strong fruity/spicy aromatic notes (such as citrus, peach, and coriander) derived from added syrups and extracts. Conversely, Sample H represents a traditional ale style (Pale Ale), featuring a robust malt backbone, pronounced hop bitterness and an absence of sweet adjuncts. The selected beers are readily available in grocery stores in Korea, and these samples are widely consumed.

**Table 1:** Main ingredients of commercial lager and ale beers.

Sample	Fermentation type	Style	ABV (%)	Ingredients
A	Bottom	Lager	4.6	Water, barley malt, hops
B	Bottom	Lager	5.0	Water, barley malt, hops
C	Bottom	Lager	5.0	Water, barley malt, sucrose, hops
D	Bottom	Lager	5.0	Water, barley malt, hops
E	Top	Ale	5.0	Water, barley malt, wheat, glucose syrup, aromatic caramel, flavorings, hop extract, coriander, sweet orange peel
F	Top	Ale	5.0	Water, wheat malt, barley malt, sugar syrup, caramel syrup, hops, flavorings, coriander seed
G	Top	Ale	4.5	Water, barley malt, wheat malt, hop pellets, yeast, natural flavorings (peach, passionfruit, pineapple), citrus extract
H	Top	Ale	4.4	Water, barley malt, hops, yeast

### 2.2 E-tongue measurement

The E-tongue (ASTREE II, Alpha MOS, Toulouse, France) was utilized to evaluate the taste attributes of the commercial canned beers. The system consists of five taste sensors, mimicking human-perceived tastes. The taste sensors of E-tongue are included in sourness (AHS), saltiness (CTS), umami (NMS), sweetness (ANS), and bitterness (SCS). Beer samples were prepared by diluting 5 mL of each sample with 95 mL of purified water in an E-tongue vial. After the preparation of the sample, the sample was loaded into the E-tongue sampler, and the taste sensors were immersed in the sample solution for 2 min to record each taste intensity. To minimize contamination between samples during E-tongue measurements, each E-tongue sensor was rinsed in purified water after analysis. The measurement was conducted with six times per sample (12).

### 2.3 E-nose measurement

The measurement of E-nose (HERACLES Neo, Alpha MOS, Toulouse, France) was utilized to evaluate volatile compounds and odor attributes in commercial canned beers. Each beer sample (5 mL) was put in an E-nose vial (22.5 × 75 mm, PTFE (polytetrafluoroethylene)/silicone septum, aluminum cap), and the vial was stirred at 500 rpm for 20 min at 40 °C for equilibrium of volatiles in the incubator. Volatile compounds in the beer were collected by the automatic sample collector of the E-nose system. Volatiles (2,000 μL) were collected and injected into the injection port. In the measurement conditions, trap absorption temperature was set 40 °C, and trap desorption temperature was set to 250 °C. Acquisition time was 110 s, and the flaw rate of hydrogen gas was 1 mL/min. Initial oven temperature was set at 40 °C for 5 s, and it ramped up to 270 °C at a rate of 4 °C/s (held for 30 s). For the identification of each volatile compound in the beer, the separated peak was identified using the retention index through AcroChemBase (Alpha MOS) library of the E-nose. The retention index of each volatile compound was identified using carbon atoms through Kovat’s index library. The measurement was conducted with triplicate samples (12).

### 2.4 Statistical analysis

The results of E-tongue and E-nose are represented as means and standard deviations. For the multivariate analysis, principal component analysis (PCA) and hierarchical cluster analysis (HCA) were performed using XLSTAT software version 2024. 4.0 (Addinsoft, New York, NY, United States) to identify the data patterns and classify the samples based on taste and odor attributes. For the PCA, the results were based on orthogonal variable sets, which are known as the principal components (PCs) based on Kaiser’s rules. PCs resulted from eigenvalues (>1.0). HCA resulted from dissimilarities among samples, and the results were represented as a dendrogram. The dissimilarities were calculated based on the Euclidean distance among samples using Ward’s algorithm as the agglomerative method. Partial least squares discriminant analysis (PLS-DA), correlation heat map, and DSPC network were performed using MetaboAnalyst 6.0^1^ (11).

## 3 Results and discussion

### 3.1 E-tongue analysis

**Figure 1:**
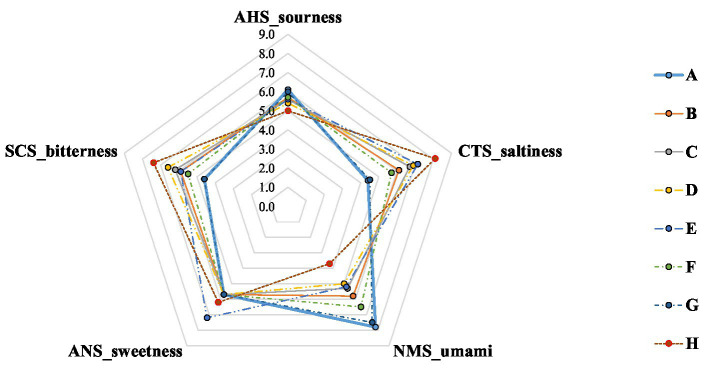
Taste attributes of commercial canned beers using E-tongue.

Taste attributes of commercial lager and ale beers were evaluated using E-tongue, and the results are shown in Figure 1. A total of eight canned beers were evaluated, and five taste sensor values were measured. Among the five taste sensors, the AHS (sourness) sensor value was the highest in the A sample and the lowest in the H sample. The CTS (saltiness) sensor value was the highest in the H sample and the lowest in the G sample. The NMS (umami) sensor value was the highest in the A sample and the lowest in the H sample. The ANS (sweetness) sensor value was the highest in the E sample, and other samples showed similar ANS values. The SCS (bitterness) sensor value was the highest in the H sample and was the lowest in the A and G samples. The E-tongue evaluates the taste patterns of food products by measuring the electrical signals of soluble taste attributes using five basic taste sensors. The exploration of taste attributes using E-tongue is developed by mimicking the human gustatory system (10, 17, 18). The gustatory perception usually plays a crucial role in the satisfaction and quality of foods. Unlike human sensory evaluation, the E-tongue has several advantages, including rapid detection, objective results, and low cost (12). In addition, E-tongue is used to discriminate food products based on their taste sensor values (11). In the E-tongue, the umami sensor is associated with concentrations of free amino acids, aspartic acids, and glutamic acids (17). The sourness and sweetness sensors are associated with hydrogen ions (H^+^) and glucose. The saltiness sensor is associated with sodium chloride (NaCl), and the bitterness sensor is associated with magnesium chloride (MgCl), tannin, and quinine (19). Regarding the taste attributes captured by the E-tongue, the observed sensor variations can be directly associated with the samples’ ingredient compositions (Table 1) and brewing conditions. In particular, the higher sweetness signal (ANS) observed in ale samples E, F, and G is likely attributable to their adjunct formulations, which is consistent with prior studies showing that E-tongue responses reflect compositional differences and can effectively discriminate samples with varying sweetener or additive contents (17, 20, 21). Unlike the standard lager samples, these commercial ales incorporated unfermented residual sugars and flavorings, such as glucose/sugar syrups, caramel, and fruit extracts (peach, passionfruit, and sweet orange peel). The prominent presence of these sweet-associated compounds likely contributed to the elevated ANS sensor responses, consistent with the evidence that adjuncts and syrup-based ingredients can alter beer sensory properties and that electronic tongue systems can discriminate beverages according to compositional differences (20, 22, 23). Conversely, Ale Sample H exhibited the highest bitterness (SCS) and saltiness (CTS) intensities, indicating a distinctly different sensory balance from the other ale samples (24). Unlike the standard lager samples, the commercial ales were characterized by the presence of residual sugars and supplementary flavoring ingredients, including glucose/sugar syrups, caramel, and fruit-derived additives. The abundance of these sweet-associated compounds may explain the elevated ANS sensor responses, in line with previous studies showing that both the beer matrix composition and fruit- or wood-derived flavor constituents can substantially modify sensory attributes (25–27). By contrast, Ale Sample H showed the highest bitterness (SCS) and saltiness (CTS), suggesting a sensory profile distinct from that of the other ale samples, which is consistent with the discriminative capacity of electronic tongue systems in beverage analysis (28). In this study, taste attributes of commercial canned beers were determined, and the measurement of E-tongue identified the five tastes of lager and ale canned beers.

### 3.2 Multivariate analysis based on E-tongue data

**Figure 2:**
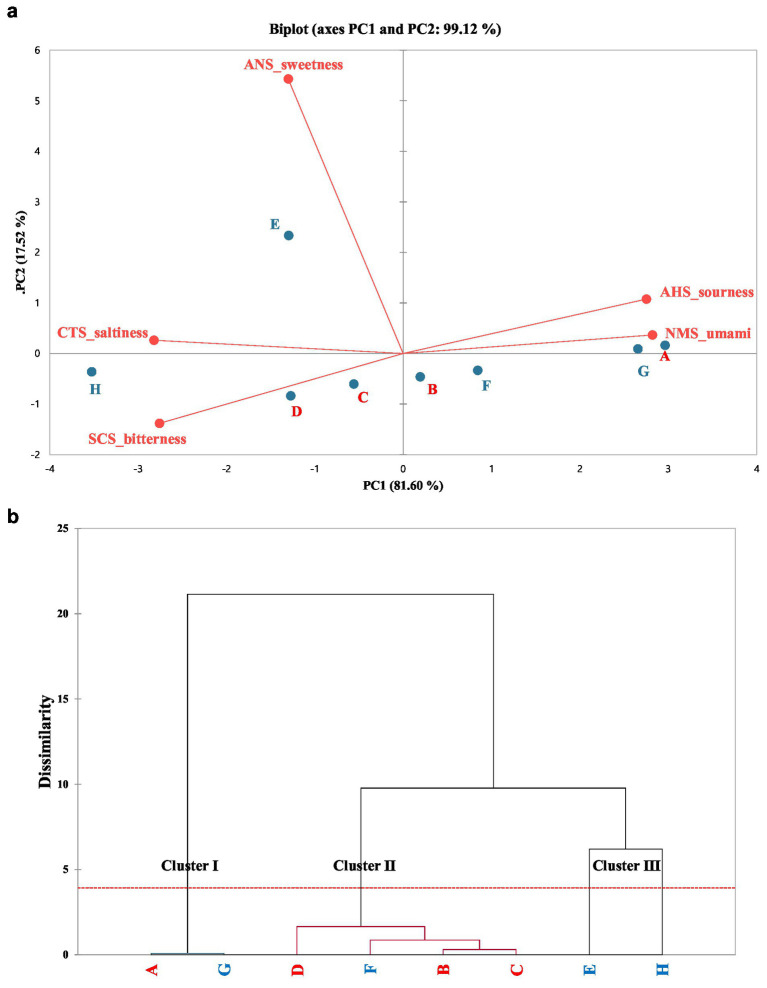
Taste patterns of commercial canned beers through multivariate analysis **(a)** principal component analysis and **(b)** hierarchical cluster analysis.

Taste patterns in commercial lager and ale beers were identified through multivariate analysis (PCA-biplot and HCA), and the results are shown in Figures 2a,b. Eight beer samples showed different taste patterns, and PC1 represented 81.60% of the variance and PC2 represented 17.52% variance. Hence, 99.12% variances have represented. Among all samples, four samples (A, B, F, and G) were located on the positive axis of PC1, and A and G samples were primarily influenced by umami (NMS) and slightly influenced by sourness (AHS). Other samples (C, D, E, and H) were located on the negative axis of PC1, and the H sample was primarily influenced by saltiness (CTS) and bitterness (SCS). The E sample was slightly influenced by sweetness (ANS). In the PC2 axis, most lager samples (B, C, and D) were located on the negative axis of PC2, and the other sample (A) was located on the positive axis of PC2. In the HCA results, a total of three clusters were identified, and A and G samples were classified as cluster 1. B, C, D, and F samples were classified as cluster 2, and E and H samples were classified as cluster 3.

Recently, the multivariate analysis has been used to classify and discriminate the food samples through the combination of logical, mathematical, and statistical methods to efficiently manage and interpret chemical datasets. In the case of the PCA and HCA, PCA is known as a useful analytical tool for compressing large chemical-derived data into summarized and visualized principal components (variables) and patterns (9, 10). HCA is known for classifying the relationships between samples based on similarity or dissimilarity, resulting in cluster(s) (10, 12). In this study, most beer samples were primarily influenced by sourness, umami, and bitterness sensors of the E-tongue system, and the three clusters were represented. Particularly, most lager beers (B, C, and D) were classified as cluster 2, and most ale beers were classified as other clusters. The spatial distribution in the PCA and the resulting HCA clusters can be directly explained by the dominant taste matrices of the studied styles (5). For example, the grouping of most lager beer samples (B, C, and D) into Cluster II reflects their shared traditional bottom-fermentation process, which yields a highly consistent, clean, and attenuated baseline profile without extreme taste deviations (4, 5). In contrast, the wider dispersion of the ale samples across different clusters (e.g., Clusters I and III) is driven by their diverse formulations. Ales with sweet adjuncts (syrups, fruit extracts) or significantly higher hopping rates create strong directional pulls in the PCA space (toward intense sweetness or bitterness), inherently separating them from the uniform lager baseline. Therefore, E-tongue has a potential application in discriminating commercial beer samples through taste patterns.

### 3.3 E-nose analysis

The volatile compound profiles in the commercial lager and ale beers were evaluated using the E-nose system, and the results are detailed in Table 2. A total of 34 volatile compounds were identified, including three acids, four aldehydes, seven alcohols, nine esters, four hydrocarbons, five heterocyclics, and three ketones. In the case of volatile acids, three volatile compounds were identified, and butanoic acid was detected in a majority of the beer samples. However, the H sample was not detected. 3-Methylbutanoic acid was only detected in the G sample. Pentanoic acid was detected in all ale beer samples and the B sample. Volatile acids in beer are commonly generated during the fermentation of beer, and they have a relatively low odor threshold (18, 19, 29).

**Table 2:** Volatile compound profiles in commercial canned beers using E-nose (peak area × 10^3^).

Volatile compounds	RT(s) (RI^a^)	Sensory description	A	B	C	D	E	F	G	H
Acids (3)										
Butanoic acid	45.93 (816)	Butter, cheese, rancid	0.09 ± 0.02	0.04 ± 0.03	0.17 ± 0.16	0.05 ± 0.04	0.07 ± 0.02	0.07 ± 0.01	0.20 ± 0.01	ND
3-Methylbutanoic acid	50.67 (862)	Acidic, cheese, rancid	ND^b^	ND	ND	ND	ND	ND	0.07 ± 0.01	ND
Pentanoic acid	54.93 (902)	Beef, cheese, sour	ND	0.13 ± 0.06	ND	ND	0.10 ± 0.03	2.01 ± 0.06	0.90 ± 0.03	0.39 ± 0.04
Aldehydes (4)										
Acetaldehyde	16.02 (438)	Fresh, fruity, pungent	0.52 ± 0.04	0.50 ± 0.08	0.53 ± 0.05	0.49 ± 0.08	0.54 ± 0.07	0.47 ± 0.04	0.27 ± 0.02	0.38 ± 0.04
3-Methylbutanal	28.39 (651)	Almond, fruity, green	ND	2.90 ± 1.56	ND	ND	1.02 ± 0.24	ND	ND	ND
2-Methylbutanal	29.27 (660)	Almond, green, malty	1.12 ± 1.30	ND	3.21 ± 1.44	ND	1.65 ± 0.48	ND	ND	ND
*α*-Terpinen-7-al	79.14 (1276)	Fatty	0.11 ± 0.01	0.07 ± 0.01	0.13 ± 0.03	0.15 ± 0.01	0.10 ± 0.01	0.14 ± 0.01	0.06 ± 0.01	ND
Alcohols (7)										
Ethanol	17.31 (466)	Alcoholic, pungent	897.89 ± 28.68	890.87 ± 58.81	840.08 ± 27.07	897.47 ± 45.00	851.99 ± 54.98	864.12 ± 51.93	720.76 ± 16.91	787.76 ± 17.56
Ethanethiol	19.63 (515)	Sulfurous	ND	ND	ND	ND	ND	10.64 ± 1.02	ND	ND
Propanol	21.34 (550)	Alcoholic, fruity, pungent	15.64 ± 2.00	16.32 ± 1.58	14.94 ± 0.81	16.33 ± 1.56	14.84 ± 1.36	15.52 ± 1.71	13.28 ± 0.86	14.65 ± 1.19
2-Methyl-1-propanol	25.90 (624)	Alcoholic	6.43 ± 2.74	9.31 ± 1.22	10.27 ± 2.00	11.30 ± 3.41	10.69 ± 0.52	16.90 ± 3.15	12.93 ± 2.65	7.18 ± 1.32
2-Methyl-1-butanol	37.15 (737)	Fruity, malty	20.81 ± 0.39	19.26 ± 0.29	17.57 ± 1.39	18.44 ± 0.68	24.28 ± 1.09	22.33 ± 1.17	29.18 ± 1.26	16.90 ± 1.48
2-Hexen-1-ol	49.88 (853)	Green, leafy, walnut	ND	ND	ND	ND	ND	ND	0.18 ± 0.04	0.07 ± 0.01
2-Undecanol	80.86 (1302)	Fruity	ND	ND	ND	ND	0.10 ± 0.01	ND	0.07 ± 0.01	ND
Esters (9)										
Ethyl acetate	24.83 (612)	Acidic, caramelized, fruity	25.43 ± 1.22	25.99 ± 1.09	22.23 ± 0.15	41.99 ± 1.658	19.69 ± 0.88	42.62 ± 2.57	20.55 ± 0.61	22.66 ± 0.97
Ethyl acrylate	32.43 (701)	Fruity	ND	1.37 ± 1.20	ND	ND	ND	ND	ND	ND
Propyl acetate	34.31 (712)	Caramelized, fermented, fruity	0.38 ± 0.03	0.88 ± 0.73	0.83 ± 0.82	0.82 ± 0.24	0.26 ± 0.02	0.57 ± 0.29	0.69 ± 0.64	0.42 ± 0.14
Ethyl butyrate	44.47 (801)	Banana, bubblegum, fruity	0.37 ± 0.01	0.24 ± 0.01	0.44 ± 0.29	0.40 ± 0.04	0.63 ± 0.03	1.40 ± 0.10	2.51 ± 0.03	0.28 ± 0.02
Isoamyl acetate	52.39 (879)	Banana, fruity, sweet	15.89 ± 0.07	9.39 ± 0.22	11.91 ± 0.13	21.95 ± 0.86	10.92 ± 0.43	24.34 ± 1.10	7.51 ± 0.15	2.61 ± 0.20
Propyl pentanoate	63.03 (1013)	–	ND	1.47 ± 0.11	1.19 ± 0.03	1.01 ± 0.05	6.40 ± 0.35	5.72 ± 0.19	6.31 ± 0.11	6.97 ± 0.21
Benzyl acetate	73.06 (1161)	Boiled vegetable, burnt, floral	ND	ND	ND	ND	ND	0.07 ± 0.01	ND	ND
Propyl nonanoate	85.81 (1397)	Fermented	0.22 ± 0.08	0.26 ± 0.08	0.53 ± 0.02	0.31 ± 0.10	0.21 ± 0.02	0.34 ± 0.09	0.40 ± 0.05	0.30 ± 0.06
Nonanoic acid, hexyl ester	100.52 (1698)	Floral	ND	ND	ND	ND	ND	ND	ND	ND
Hydrocarbons (4)										
*α*-Pinene	58.64 (950)	Herbaceous	ND	ND	ND	ND	0.99 ± 0.03	0.11 ± 0.01	1.50 ± 0.07	ND
Limonene	65.99 (1045)	Citrus, fruity, mint	ND	ND	0.06 ± 0.01	ND	95.19 ± 2.66	75.82 ± 1.34	2.52 ± 0.12	0.17 ± 0.06
Terpinolene	67.68 (1074)	Fruity, herbaceous, woody	ND	ND	ND	ND	0.86 ± 0.12	11.42 ± 0.29	0.16 ± 0.01	ND
*β*-Caryophyllene	90.82 (1498)	Fruity, green, woody	ND	ND	ND	ND	ND	0.15 ± 0.03	ND	ND
Heterocyclics (5)										
Trimethylamine	14.68 (411)	Fishy, pungent	0.10 ± 0.01	0.09 ± 0.01	0.10 ± 0.01	0.11 ± 0.02	0.09 ± 0.01	0.11 ± 0.02	0.09 ± 0.01	0.09 ± 0.01
Pyrrole	39.61 (761)	Nutty	0.09 ± 0.08	0.22 ± 0.17	ND	0.09 ± 0.01	ND	0.16 ± 0.01	ND	0.79 ± 0.08
Dimethyl trisulfide	60.05 (968)	Cabbage, onion, sulfurous	ND	ND	ND	ND	ND	0.24 ± 0.02	ND	ND
2-Isopropyl-3-methoxy pyrazine	70.41 (1105)	Bean, bell pepper, green	0.23 ± 0.03	0.31 ± 0.02	0.31 ± 0.13	0.28 ± 0.03	1.10 ± 0.11	7.22 ± 0.19	0.64 ± 0.04	0.49 ± 0.04
2-Pentyl pyridine	75.05 (1197)	Fatty	1.68 ± 0.09	1.41 ± 0.06	2.09 ± 0.04	2.49 ± 0.13	1.60 ± 0.06	1.86 ± 0.17	3.45 ± 0.17	1.21 ± 0.04
Ketones (2)										
1-Hexen-3-one	41.39 (775)	Cooked vegetable, leafy	0.26 ± 0.02	0.22 ± 0.02	0.75 ± 0.67	0.36 ± 0.03	0.49 ± 0.04	1.55 ± 0.08	0.37 ± 0.07	ND
*β*-Ionone	88.35 (1465)	Floral, woody	ND	ND	ND	ND	ND	0.14 ± 0.01	ND	0.09 ± 0.01

a RI: retention index.

b ND: not detected.

In the case of the volatile aldehydes, four volatile compounds were identified. Among all aldehydes, acetaldehyde was detected in all beer samples, and *α*-terpinen-7-al was detected in most beer samples, regardless of the H sample. 3-Methylbutanal was detected in B and E samples, and 2-methylbutanal was detected in Samples A, C, and E. Volatile aldehydes are commonly generated through the oxidation of fatty acids and/or amino acids (30).

Among the volatile alcohols, seven volatile compounds were identified. Ethanol exhibited the highest peak area among all volatile compounds, with lager beers showing relatively higher peak areas than ale beers. In addition, propanol, 2-methyl-1-propanol, and 2-methyl-1-butanol were also detected in all beer samples. Among these alcohols, propanol showed relatively higher concentrations in most lager beers compared with ale beers. 2-methyl-1-propanol and 2-methyl-1-butanol showed relatively higher concentrations in most ale beers compared with lager beers. Volatile alcohol is commonly generated during yeast metabolism, and it is produced as by-products of amino acid synthesis from pyruvate via the anabolic pathway or catabolism. Alcohol in beer is present in abundant concentration and play a key role in pungent and strong flavor attributes (31).

In the case of the volatile esters, nine volatile compounds were identified. Ethyl acetate showed the highest peak area among volatile esters, followed by isoamyl acetate and propyl pentanoate. Isoamyl acetate showed the second-highest peak area among esters. Propyl pentanoate showed a relatively higher peak area in ale beers compared with lager beers. Volatile ester is known to be present in high concentrations in beer and are commonly generated from esterification of carboxylic acids and alcohols during alcoholic fermentation. This compound in beer is associated with the growth of yeast and lipid metabolism and is generally associated with fruity and sweet odor descriptions (31, 32).

In the case of volatile hydrocarbons, four volatile compounds were identified. Among all hydrocarbons, *α*-pinene and terpinolene were only detected in ale beer samples. Limonene was detected in all ale beer samples and also detected in the C sample. Volatile hydrocarbons are commonly found in food products; however, they have a higher odor threshold than other volatiles (29, 32). In this study, limonene was only found in the C sample (the lager beer); however, limonene was not found in other lager beers. In addition, other volatile hydrocarbons were not found in most lager beers. These results suggested that volatile functional groups can serve as important indicators reflecting the distinct volatile profiles (volatile hydrocarbons) between these commercial lager and ale beers.

For heterocyclics, five volatile compounds were identified. Among heterocyclics, trimethylamine, 2-isopropyl-3-methoxy pyrazine, and 2-pentyl pyridine were detected in all beer samples. 2-Pentylpyridine showed a relatively higher peak area in most beer samples except for the F sample. Volatile heterocyclic compounds are responsible for the unique odor description, and they have been found in foods (33).

In the case of ketones, two volatile compounds were identified. 1-Hexen-3-one was detected in most beer samples except for the E sample. *β*-Ionone was only detected in ale beers (I and L). Volatile ketones are commonly generated from lipid oxidation during fermentation, and this compound was found in beer samples (4, 34). The distinct volatile patterns observed between the lager and ale beers can be fundamentally explained by the metabolic divergence of their respective yeast strains and the associated brewing parameters (5). The elevated concentrations of volatile esters and higher alcohols in the ale samples are closely associated with the top-fermentation characteristics of *Saccharomyces cerevisiae*, which typically operates at warmer temperatures (15–25 °C) (5, 31). These higher temperatures accelerate yeast growth and metabolic kinetics, upregulating the activity of alcohol acetyltransferase (AATase) enzymes, which directly drive the intensive synthesis of fruity esters, such as ethyl acetate and isoamyl acetate (31). In contrast, the cleaner, more attenuated volatile fingerprint of the lager samples is a direct result of the bottom-fermentation traits of *Saccharomyces pastorianus* (4, 5). Lager brewing involves prolonged cold fermentation (7–15 °C) and subsequent cold conditioning (4). This low-temperature environment slows down lipid metabolism and suppresses the overproduction of heavy esters and organic acids, yielding the characteristically crisp., balanced, and subtle taste-masking profile typical of lager styles (4, 31). In this study, the absence of trace hop-derived odorants (e.g., monoterpenoid alcohols and thiols) in the E-nose analysis is primarily due to the system’s higher detection thresholds compared to targeted techniques such as SPME-GC-MS (4). Consequently, these trace compounds are likely masked by abundant fermentation volatiles such as ethanol and esters (31). In addition, these highly volatile hop aromas may have degraded during the distribution and extended shelf-life of the commercial canned beers.

### 3.4 Multivariate analysis based on E-nose data

**Figure 3:**
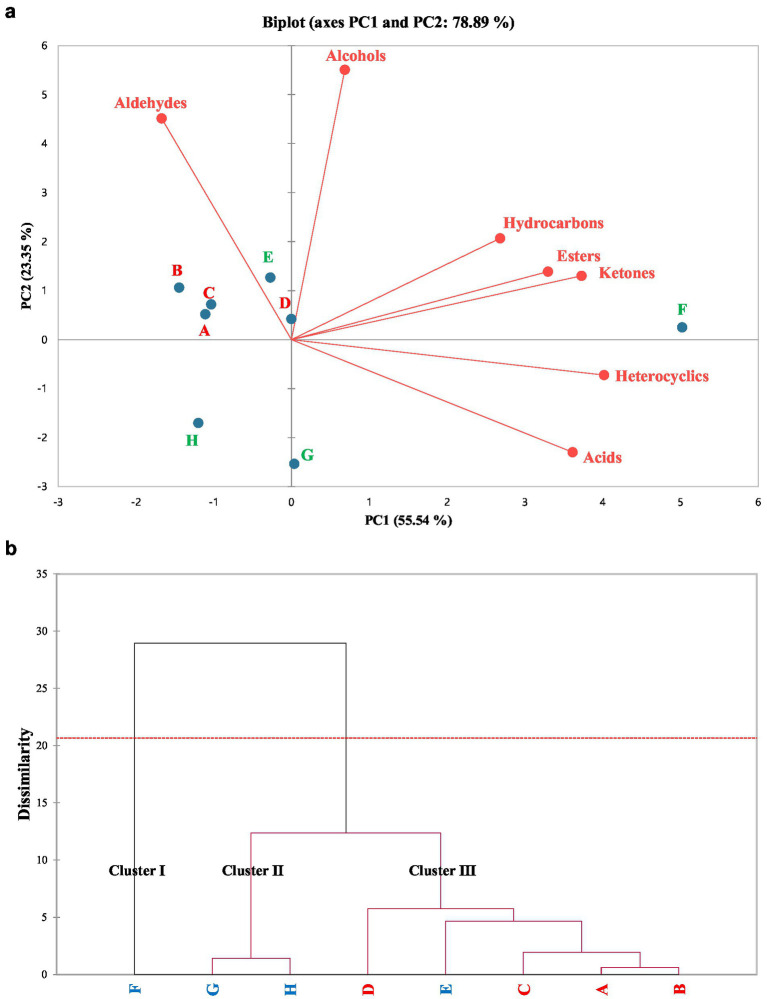
Odor patterns of commercial canned beers through multivariate analysis **(a)** principal component analysis and **(b)** hierarchical cluster analysis.

Odor patterns of volatile compound profiles in commercial lager and ale beers were identified through multivariate analysis (PCA-biplot and HCA), and the results are shown in Figures 3a,b. Eight beer samples showed different odor patterns, and PC1 represented 55.54% variance, and PC2 represented 23.35% variance. Hence, 78.89% variances have represented. In the PC1 axis, most beer samples were located on the negative axis of PC1, and others (F and G) were located on the positive axis of PC1. Particularly, the F sample was mainly influenced by heterocyclics and ketones and slightly influenced by acids, esters, and hydrocarbons. In the PC2 axis, most beer samples were located on the positive axis of PC2, and G and H samples were located on the negative axis of PC2. In the HCA results, three clusters were identified, and the F sample was classified into cluster 1. G and H samples were classified as cluster 2, and A–E samples were classified as cluster 3.

In this study, most beer samples were mainly separated according to lager and ale beer style. Particularly, the F sample showed distinct odor patterns influenced by esters, ketones, and heterocyclics. The E-nose was developed to mimic the responses of mammals to olfactory stimuli to odors released by the volatile compounds, and odor characteristics in foods with odor characteristics of weak selectivity, non-specific determination, and cross-sensitivity (17). In addition, the E-nose is widely used to discriminate the odor attributes and examine the food odors (8–10). Previous studies have successfully utilized E-nose platforms combined with multivariate analysis to discriminate complex odor profiles in various food matrices, such as citrus peels (30) and hydrolyzed food by-products (35). Building upon these established methodologies, our PCA and HCA results demonstrated that the volatile odor patterns (E-nose) exhibited a much more pronounced spatial separation between the lager and ale styles compared to the non-volatile taste patterns (E-tongue). This finding suggests that yeast-driven volatile synthesis plays a more decisive role in beer style differentiation than the baseline taste attributes. In this study, odor patterns were investigated using PCA and HCA. The separation observed in the multivariate odor patterns is fundamentally driven by metabolic differences among the yeast strains utilized (5). The divergence of specific ale samples (such as F–H) from the primary lager cluster is likely attributed to their elevated ester, ketone, and higher-alcohol profiles (4). This clustering pattern is a direct biochemical consequence of the top-fermentation process by *Saccharomyces cerevisiae* at warmer temperatures (15–25 °C), which significantly upregulates ester-synthesizing enzymes (e.g., alcohol acetyltransferase, AATase) (5, 31). Conversely, the tighter grouping of traditional lager beers reflects the suppressed volatile synthesis characteristic of cold bottom-fermentation (*Saccharomyces pastorianus*), which inherently produces a cleaner, less ester-driven aromatic profile, causing them to cluster closely together in the PCA space (4, 31).

Overall, the results of this study indicated that odor patterns (E-nose) showed relatively higher variations compared with taste patterns (E-tongue) according to lager and ale beers.

### 3.5 Multivariate analysis based on the combination between E-tongue and E-nose data

**Figure 4:**
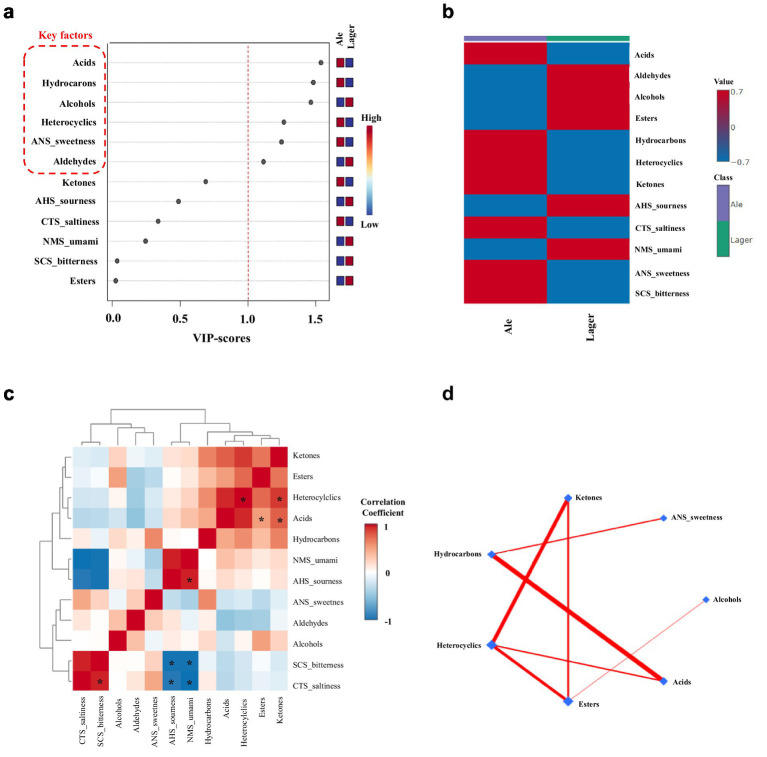
Key flavor attributes and interaction between tastes and volatiles in commercial canned beers through multivariate analysis **(a)** variable importance in projection, **(b)** fingerprints of flavor attributes between lager and ale beers, **(c)** correlation heatmap, **(d)** debiased sparse partial correlation network. * Means significantly different from 0 at alpha = 0.05.

PLS-DA (VIP-scores), correlation heatmap, and DPSC network were performed to investigate the combination data of E-tongue and E-nose results, and the results are shown in Figures 4a–d. In VIP-scores (Figure 4a), a total of six significant contributors (key factors) were identified, including acids, hydrocarbons, alcohols, heterocyclics, sweetness, and aldehydes. In detailed, three volatile and one taste attributes of VIP-scores were higher than 1 (VIP score ≥1). The fingerprints of flavor attributes between lager and ale beers were identified using the clustering heatmap based on VIP-scores (Figure 4b), and lager and ale beers showed the different key variables. In correlation heatmap results (Figure 4c), the correlation between taste attributes (E-tongue) and odor attributes were examined through Pearson’s correlation analysis. The significant levels (*p* < 0.05) of correlation were identified. Heterocyclics showed positive correlates with acids (0.946), ketones (0.904), and esters (0.737), and ketones also showed positively correlated with acids (0.780). Umami and bitterness showed positively correlated with sourness (0.966) and saltiness (0.962), respectively. On the contrary, umami and bitterness showed negatively correlated with saltiness (−0.993) and umami (−0.982), respectively. In addition, saltiness and bitterness showed negatively correlated with sourness (−0.948 and −0.979). In DSPC network (Figure 4d), a total of seven nodes were identified, including ketones, hydrocarbons, heterocyclics, esters, acids, alcohols, and sweetness. The relationship between taste attributes and odor attributes were examined, and the positive coefficient was represented by red lines.

PLS-DA is commonly utilized to classify and discriminate the data, and it combines regression models to reduce the number of dimensions and variables (or features) to a certain threshold to categorize the results of regression. VIP-score is associated with most significant contributors (important features) among variables (volatile compounds and taste attributes), and it is calculated to determine the key flavor attributes in this study. A VIP score of ≥1 indicates potential key marker(s), and a higher VIP score represents a more significant difference among significant contributors (12). Previous studies have reported that sensory cognition was primarily influenced by the combination of flavor compounds rather than single taste and/or volatile compounds (36–38). In addition, the differences in flavor patterns in lager and ale beers according to different types of fermentation have been identified (4, 5, 14). The results of this study also identified differences in flavor patterns between commercial lager and ale beers. Pearson correlation is widely used to measure the statistical relationships between random features (variables) (39). DSPC network is a useful model for exploring connectivity correlations and patterns, and it is based on the Gaussian graphical modeling algorithm. The DSPC network can be utilized with a small number of samples, and this model calculates partial correlations to remove indirect relationships in the DSPC network, generating a set of potential interactions with high confidence and minimizing false positives (30). In this study, key flavor compounds and attributes between lager and ale beers were identified through VIP-scores (PLS-DA), and the interactions between taste and volatile compounds were identified through the correlation heatmap and DSPC network according to the fermentation types of commercial canned beers (lager and ale beers).

## 4 Conclusion

This exploratory study demonstrated the feasibility of utilizing E-tongue and E-nose platforms combined with chemometrics to differentiate the specific profiles of commercial lager and ale beers. For E-tongue analysis, the sensor values of five tastes were measured, and eight beer samples showed variations of taste attributes. For E-nose analysis, three acids, four aldehydes, seven alcohols, nine esters, four hydrocarbons, five heterocyclics, and two ketones were identified among all beer samples, and alcohols and esters showed relatively higher peak areas compared with other functional groups of volatile compounds. For the multivariate analysis, taste patterns were investigated through PCA, and three clusters were identified through HCA. In particular, a majority of the lager beers were classified as cluster II. Odor patterns were investigated through PCA, and three clusters were identified through HCA. A majority of the ale beers were classified as clusters I and II, and lager beers were classified as cluster III. Key flavor compounds were identified through VIP-scores, and six compounds (VIP score ≥ 1), including acids, hydrocarbons, alcohols, heterocyclics, sweetness, and aldehydes, were identified as the significant contributors. The differences between lager and ale beers were identified through heatmap clustering based on taste attributes and volatile compounds and attributes. A correlation heatmap revealed six positive and four negative interactions between the taste and odor attributes. Furthermore, the DSPC network analysis identified seven key flavor variables, including ketones, sweetness, alcohols, acids, esters, heterocyclics, and hydrocarbons. Despite the limitations of a small sample cohort and potential confounding product factors, the primary innovation of this study has been successfully demonstrated. The integrated E-sensing framework, particularly when combined with DSPC network analysis, provides a valuable pilot approach for rapid, non-destructive screening in food-tech applications. Furthermore, a notable limitation of this preliminary study is the absence of parallel traditional instrumental quantification (e.g., GC-MS, HPLC) and human sensory panel evaluations. Therefore, the direct association between the E-sensor outputs, absolute compound concentrations, and actual human perception cannot be definitively established. Further research should focus on validating these E-sensing fingerprints by correlating the sensor responses with targeted chromatographic analyses and descriptive sensory profiling. Ultimately, this methodology offers a promising, objective, and cost-effective alternative to traditional sensory panels for routine quality control and flavor fingerprinting in the commercial brewing industry.

## Data Availability

The original contributions presented in the study are included in the article/supplementary material, further inquiries can be directed to the corresponding author.

## Footnotes

1
https://www.metaboanalyst.ca
